# Time to death and its determinants among under-five children in Sub-Saharan Africa using the recent (2010–2018) demographic and health survey data: country-based shared frailty analyses

**DOI:** 10.1186/s12887-021-02950-3

**Published:** 2021-11-17

**Authors:** Desalegn Tesfa, Sofonyas Abebaw Tiruneh, Melkalem Mamuye Azanaw, Alemayehu Digssie Gebremariam, Melaku Tadege Engdaw, Belayneh Kefale, Bedilu Abebe, Tsion Dessalegn

**Affiliations:** 1 Department of Public Health, College of Health Sciences, Debre Tabor University, Debre Tabor, Ethiopia; 2 Department of Pharmacy, College of Health Sciences, Debre Tabor University, Debre Tabor, Ethiopia

**Keywords:** Survival status, Under-five mortality, Determinant factors, Shard frailty, Sub-Saharan Africa

## Abstract

**Background:**

Substantial global progress has been made in reducing under-five mortality since 1990, yet progress is insufficient to meet the sustainable development goal of 2030 which calls for ending preventable child deaths. There are disproportional survivals among children in the world. Therefore, the study aimed to assess the Survival status of under-five mortality and determinants in Sub-Saharan African Countries using the recent DHS data.

**Methods:**

The data was retrieved from the birth record file from the standard Demographic and Health Survey dataset of Sub-Saharan Africa countries. Countries that have at least one survey between 2010 and 2018 were retrieved. Parametric shared frailty survival analysis was employed.

**Results:**

A total of 27,221 (7.35%) children were died before celebrating their fifth birthday. Children at an early age were at higher risk of dying and then decrease proportionally with increased age. The risk of death among rich and middle family were lowered by 18 and 8% (AHR =0.82, 95% CI: 0.77-0.87) and (AHR = 0.92, 95% CI: 0.87-0.97) respectively, the hazard of death were 11, 19, 17, 90 and 55% (AHR = 1.06, 95% CI: 1.00-1.12), (AHR = 1.11,95%CI:1.04-1.19), (AHR = 1.17, 95% CI:1.12-1.23), (AHR = 1.90, 95%CI: 1.78-2.04) and (AHR = 1.55, 95% CI:1.47-1.63) higher than among children in rural, use unimproved water, delivered at home, born less than 18 months and between 18 and 23 months birth intervals respectively. The hazard of death was 7% among females and low birth weights (AHR = 0.93, 95%CI: 0.90 – 0.97) and (AHR = 0.93 95%CI: 0.89-0.97) respectively. There was also a significant association between multiple births and birth orders (AHR = 2.11, 95%CI: 2.51 – 2.90), (AHR = 3.01, 95%CI: 2.85-3.19) respectively.

**Conclusions:**

Death rate among under-five children was higher at an early age then decreases as age advanced. Wealth status, residence, water source, place of delivery, sex of the child, plurality, birth size, preceding birth interval, and birth order were the most predictor variables. The health care program should be designed to encourage a healthy family structure. The health care providers should intervene in the community to inspire maternal health services.

## Background

Under-five mortality (U5MR) is a key global indicator of child health and one of the most important measurements of nation development [1–3]. It is a serious issue for demographers and public health specialists and it is a core indicator of the development of families, societies, and the earth at large [4]. Under-five mortality is the probability of dying before celebrating 5 years of age and it is expressed as mortality per 1000 live birth [2, 3]. According to the report of the World Health Organization (WHO) and UNICEF [5, 6], substantial global progress has been made in reducing under-five mortality faster than any other time during the past two decades and since the 1990s, the global U5MR has dropped 53% from 91 per 100 live births in 1990s to 43 in 2015. Despite these gains, child existence remains an urgent concern. In 2013, around 17,000 children under age five die every day which showed that progress has been insufficient [7]. There are a disproportionate number of deaths among children less than 5 years of age in the world [3], particularly SSA regions had the highest death rate for children less than 5 years of age, with most of these deaths, occurred in the rural areas [3]. Because greater than two-third of the people are rural inhabitants [7–9]. Every year millions of children under 5 years of age die, mostly from preventable causes [3]. In 2015, more than 80% of the total 5.9 million under-five deaths were estimated to have occurred mostly from easily avoidable causes like pneumonia, diarrhea, and malaria in developing countries [10]. According to an estimate from GDB 2017 collaborates, even though numerous countries are on the track for achieving the target of SDGs at least 25 death per 1000 live birth by 2030, African countries would need to increase the annual rate of decline from 2015 to 2030 that two to ten times higher than what was recorded between 1990 to 2015 to meet the stated goals [11]. Furthermore, recent studies conducted in East Africa (Rwanda, Burundi, and Tanzania) have indicated that child survival has been influenced by community socio-demographic factors, maternal and child health characteristics, environmental and behavioral factors [12]. U5MR is still highest in the WHO African countries (greater than 80 per 1000 live birth, about 7 times higher than the WHO European region 11 per 100 live births in high-income countries however it is 11 times in low-income countries [12]. In the years, between 2016 to 2030 about 95 million children are projected to die between birth and exactly 5 years of age if the 2015 mortality rate remains constant in each country, and around 70 million would die if each country continues to reduce its mortality rate at peace estimate from 2000 to 2015. If all countries achieve the SDGs of 25 or fewer death per 1000 live birth by 2030, and 56 million death by 2030, therefore about two-third of all SSA countries need to accelerate progress to achieve this goal [13]. Here, the objective of this study was to estimate time to death, and its determinants among under-five children in SSA countries which are important for concerned bodies and strategists to take instantaneous actions. And it is also important to develop different designs to achieve the front SDGs. By 2030, SDGs agree to end preventable deaths on newborns and children under 5 year’s age. Deaths happening before celebrating the fifth birthday remain an enormous public health concern, especially in SSA and South Asian countries, and an overwhelming majority (80%) of the world’s estimated under-five deaths occurred in these two sub-regions [14]. Therefore to succeed in the SDGs target, justifiable efforts are needed mainly in developing countries.

## Methods

### Data sources

The data source was the recent (2010-2018) demographic and health survey (DHS) among 33 Sub-Saharan Africa countries. This standard Demographic and Health Survey is nationally representative and population-based surveys collected through uniform questionnaires and comparable across countries. The data were collected by multi-stage stratified cluster sampling design for each country. The details of the recorded data were accessed at www.measuredhs.com.

### Populations and samples

The source population was all live births under 5 years of children preceded 5 years before each survey among 33 Sub-Saharan Africa countries. The data were extracted from the birth record (BR file) file from the standard DHS dataset of Sub-Saharan Africa countries with at least one survey from 2010 to 2018. A total of 370, 237 under-five children were included from 11 east African, 6 central African, 13 West African, and 3 South African countries.

### Eligibility identification

In this study, all live births followed for 5-year full cohort preceding 5 years the survey in the selected enumeration areas in 33 Sub-Saharan African countries. However, some Sub-Saharan African Countries such as Central Africa Republic, Eswatini, Sao Tome Principe, Madagascar, and Sudan were not included in this study because they have no recent DHS data (no report after 2010/2011). As well, three Sub-Saharan Africa Countries such as Botswana, Mauritania, and Eritrea were excluded because their data set was not publicly available.

#### Outcome variable

The outcome variable of this study was the time to under-five children’s death. The survival time of a child beyond 59 months was declared as censored whereas children who died up to 59 months were declared as an event.

#### Independent variables

The independent variables were summarized as socio-demographic characteristics (parental educational status, wealth index of the household, residence, water source, sub-region, and County income), obstetric characteristics (birth interval, place of delivery, antenatal care visit, parity, and birth order), and under five-children and maternal characteristics (sex, weight, and plurality), etc.

### Modeling of parametric shared frailty survival analysis

The frailty model is a random-effects model which has an unobserved multiplicative effect on the hazard rate for the entire individual in the same group. In the shared frailty model children in the same Country share the same nuisance (frailty) factor. Parameter *θ* provides information on the variability (the dependency) in the population in the same Country. Under-five children in the same Country i with u_i_ > 1 and u_i_ < 1 have a more frail than high risk and lower risk respectively. Based on the different frailty terms one frailty term was employed using Country taken as random effect dependency. For single frailty term model specification will be given by [15]

$${\mathrm{h}}_{\mathrm{i}\mathrm{j}\left(\mathrm{t}\right)}={\mathrm{h}}_0\left(\mathrm{t}\right){\mathrm{u}}_{\mathrm{i}}\exp \left({\mathrm{x}}_{\mathrm{i}\mathrm{j}}^{\mathrm{t}}\upbeta \right).$$

Where u_i_ = exp(wi) is called the frailty for the i^th^ Country. The u_i_’s, i = 1. . . s, are the actual values of a sample from a density *f*_*U*_.

Parametric frailty model fitted using Gompertz baseline hazard distributional assumption and gamma frailty distribution model fitted in Country taken as random effects frailty for the independent variables.

### Level of dependence in the shared frailty model

The correlation between any two event times from the same country is measured by Kendall’s tau (τ). Kendall’s tau (τ) measured the dependency of two events in the same country which is dividing the frailty (*θ*) by two-plus frailty (*θ*). The higher frailty (*θ*) leads to the higher dependency and the higher Kendall’s tau (τ).

$$\mathrm{Kendall}'\mathrm{s}\ \mathrm{tau}\ \left(\uptau \right)=\frac{\theta }{\theta +2},\mathrm{where}\ \uptau\ \upvarepsilon\ \left(0,1\right)$$

### The best fit model selection

The best fit model was selected using Akakian Information Criteria (AIC) and the Log-likelihood ratio test. The lowest Akakian Information Criteria and the highest Log-likelihood ratio value declare the best fit model. Besides, the Cox-Snell residual plot was also employed for model adequacy. If the model fits, the Cox-Snell residuals should have a standard exponential distribution with λ = 1. One way to verify the fit is to calculate an empirical estimate of the cumulative hazard function based on the Kaplan–Meier survival estimates taking the Cox–Snell residuals as the time variable. If the model fits the data, the plot should be a straight line with a slope of 1.

## Results

### Weighted and unweighted samples in SSA using demographic and health survey data

Thirty-three SSA countries were included in this study. Greater than two in five 154,897 (41.83%) of the participants were found in the West African region with one in ten 34,433 (9.30%) concentrated in Nigeria. Greater than one –third 124,908 (33.75%) of the respondents were located in the East Africa region. Around one in fifteen 21,090 (5.7%) respondents were found in Kenya (Table 1).

**Table 1:** Country-based weighted and unweighted samples in SSA using the recent (2010-2018) Demographic and Health Survey data, 2021

Country	Year of DHS	Frequency		Unweighted percent
		Unweighted	Weighted	
***East Africa region***				
**Burundi**	2016/17	13,005	13,426	3.51
**Ethiopia**	2016	10,686	11,039	2.89
**Kenya**	2014	21,090	19,693	5.70
**Comoros**	2012	3170	3254	0.86
**Malawi**	2015/16	16,942	17,026	4.58
**Mozambique**	2011	11,241	11,874	3.04
**Rwanda**	2014/15	7852	8001	2.12
**Tanzania**	2015/16	9982	9796	2.70
**Uganda**	2016	15,587	15,314	4.21
**Zambia**	2018	9559	9431	2.58
**Zimbabwe**	2015	5794	6064	1.56
***Central Africa region***				
**Angola**	2015/16	14,184	13,219	3.83
**Democratic Republic of Congo**	2013/14	18,656	18,329	5.04
**Republic of Congo**	2011/12	9209	8007	2.49
**Cameroon**	2011	11,830	11,847	3.20
**Gabon**	2012	6081	5129	1.64
**Chad**	2014/15	18,664	18,688	5.04
***South Africa region***				
**Lesotho**	2014	3183	3154	0.86
**Namibia**	2013	5075	4829	1.37
**South Africa**	2016	3550	3565	0.96
***West Africa region***				
**Burkina Faso**	2010	15,309	15,647	4.13
**Benin**	2017/18	13,472	13,520	3.64
**Ivory Coast**	2011/12	7817	7527	2.11
**Ghana**	2014	5940	5751	1.60
**Gambia**	2013	8105	7926	2.19
**Guinea**	2018	7996	7926	2.16
**Liberia**	2013	7663	6549	2.07
**Mali**	2018	10,045	10,418	2.71
**Nigeria**	2018	34,433	34,717	9.30
**Niger**	2012	12,655	13,457	3.42
**Sierra Leone**	2013	12,166	12,440	3.29
**Senegal**	2010/2011	12,320	11,443	3.33
**Togo**	2013/14	6976	6705	1.88

### Socio-demographic characteristics of the study participants

The majority 278,395 (75%) of the mothers were between the age of 20-35 years and their mean age was 29.24 ± 0.03. less than one-fourth 93,277 (24.54%) of the women were attended their secondary school and above. Around five out of seven 251,011 (70.11%) of the respondents have dwelled in rural. Nearly half 162,726 (47.75%) of the study participants had poor wealth status. Among four sub-regions of Sub-Saharan Africa: about four in ten 154,023 (42%) of them reside in the central region. However, South Africa accounts for 11,549 (3.19%) (Table 2).

**Table 2:** Socio-demographic characteristics of the study participants in Sub-Saharan Africa using the recent (2010-2018) DHS data, 2021

Variable	Category	Frequency		Unweighted percentage
		Unweighted	Weighted	
**Maternal age**	15-19	23,110	22,319	6.24
	20-35	278,395	276,459	75.20
	36-49	68,732	66,931	18.56
**Maternal age at birth**	≥20 years	309,371	306,321	83.56
	< 20 years	60,866	59,388	16.44
**Marital status**	Not currently married	22,626	21,483	6.11
	Married	347,611	344,226	93.89
**Maternal educational status**	No education	156,494	151,422	42.27
	Primary	122,889	121,010	33.19
	Secondary and above	90,854	93,277	24.54
**Husband education status**	No education	122,858	119,951	38.86
	Primary	87,816	88,112	27.77
	Secondary and above	105,510	105,710	33.37
**Husband occupational status**	No working	10,890	10,646	3.44
	Working	305,732	04,354	96.56
**Residence**	Urban	110,659	114,698	29.89
	Rural	259,578	251,011	70.11
**Sex of the household head**	Male	293,821	292,826	79.36
	Female	76,416	72,883	20.64
**Wealth status**	Poor	176,794	162,726	47.75
	Middle	72,855	73,625	19.68
	Rich	120,588	129,358	32.57
**Toilet facility**	Had toilet	42,921	247,866	65.63
	No toilet	127,194	117,712	34.37
**Water source**	Improved	83,191	86,186	22.47
	Unimproved	286,985	279,462	77.53
**Country income**	Low income	212,176	213,407.	57.31
	Lower middle income	135,359	130,853	36.56
	Higher middle income	22,702	21,449	6.13
**Sub-Saharan region**	East Africa	124,908	124,918.	33.74
	West Africa	78,624	75,219	21.24
	Central Africa	154,897	154,023	41.84
	Southern Africa	11,808	11,549	3.19

### Under five-children and maternal obstetric characteristics of the study participants

Of the total under-five children, the proportion of male and females children are almost equal (50%). Surprisingly around two-thirds, 219,360 (63.32%) of them were exposed to low birth weight and greater than half 207,429 (56.07%) of their birth order was less than three. Only 218,559 (59.15%) of the women have at least one ANC follow-up (Table 3)*.*

**Table 3:** Under-five children and maternal obstetric characteristics of the study participants in Sub-Saharan Africa using the recent (2010 -2018) DHS data, 2021

Characteristics	Category	Frequency		Unweighted percentage
		Unweighted	Weighted	
**Sex**	Male	187,282	185,107	50.58
	Female	182,955	180,602	49.42
**Plurality**	Single	356,644	352,199.	96.33
	Multiple	13,593	13,510	3.67
**Preceding birth interval**	≥ 24 months	232,453	229,021	80.24
	18-23 months	37,156	36,286	12.83
	< 18 months	20,091	19,485	6.94
**Preceding birth interval**	≥ 24 months	232,453	229,022	80.24
	< 24 months	57,247	55,770	19.76
**Birth order**	<three	207,592	207,429	56.07
	≥ four	162,645	158,280	43.93
**Birth size at birth**	Low birth weight	221,658	219,360	63.32
	Not low birth weight	128,411	127,222	36.68
**ANC follow up**	No ANC visit	151,249	147,150	40.85
	At least one ANC visit	218,988	218,559	59.15
**Place of delivery**	Health facility	226,838	228,155	61.79
	Home	140,269	134,373	38.21
**Mode of delivery**	SVD	352,688	347,167	95.26
	Cesarean section	17,549	18,542	4.74

### Under-five children’s survival in Sub-Saharan Africa

Of the total of 370,237 under-five children, 27,221 (7.35%) were died before celebrating their fifth birthday. From the total of under-five children followed for 5 years, the cumulative probability of survival at the end of 5 years was 89.54% (95% CI: 0.89-0.90). But the cumulative survival among male and female was 88.74% (95% CI: 0.89-0.90) and 90.36% (95% CI: 0. 90-0.91) respectively. The cumulative probability of survival at 1 year was 92.59% (0.92-0.93) (Table 4).

**Table 4:** Life time table cumulative survival probability of under five children in Sub-Saharan Africa using the recent (2010 - 2018 DHS data, 2021

Cumulative survival probability (95% CI)			
Time after birth	Male	Female	Total
**0-12 months**	93.46 (93. 34-93.58)	94.70 (94.59-94.80)	94.07 (93.99-94.15)
**13-24 months**	91.98 (91.84- 92.11)	93.22 (93.09-93.34)	92.59 (92.50-92.68)
**25-36 months**	90.68 (90.53-.9083)	91.98(91.84-92.13)	91.33 (91.22-91.43)
**37-48 months**	89.78 (89.61- 89.94	91.15(90.98-91.31)	90.45 (90.34-90.57)
**Five year**	88.74(88.51-88.96)	90.36(90.16-90.57)	89.54(89.39-89.69)

### Kaplan Meier survival analysis

**Fig. 1:**
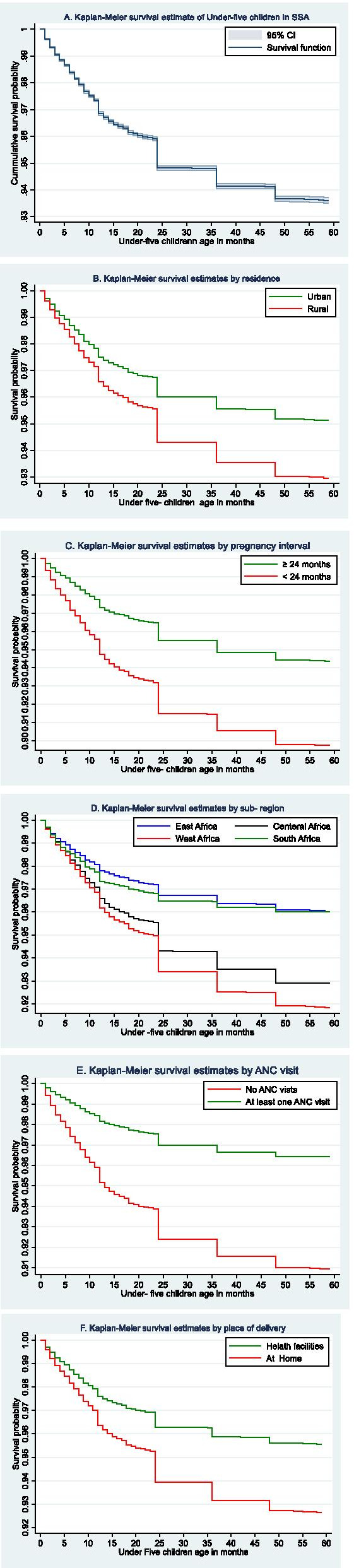
Overall survival estimate of under-five children (**A**), survival estimates in residence (**B**), survival estimates in Pregnancy interval (**C**), survival estimates in SSA-regions (**D**), survival estimates by ANC visit (**E**), survival estimates by place of delivery (**F**)

The survival probability of under-five children was estimated using Kaplan Meier survival estimate. The Kaplan Meier confirms with the life table results. The survival probability is very low among newborns and starts to stabilize when the child reaches 2 years of age. This means that children age between 0 and 2 years are at a higher risk of dying and the risk decreases proportionally with the increase of age for both children aged between 0 and 2 years and those aged between 2 and 5 years (Fig. 1A). The probability of death among children who had lower than 24 months of pregnancy interval (Fig. 1C) and women’s didn’t expose to antenatal care follow-up (Fig. 1E) were higher. The highest probability of death among under-five children was in the Western Africa region of SSA. Among those who are between the age of one and 2 years old children, the probability of death was higher among children living in South Africa. However, the survival probability of under-five children after 2 years of age in West Africa and Central Africa was proportional through 5 years of life. Similarly, the probability of survival of children after 2 years was proportional throughout 5 years period of life in East and South Africa (Fig. 1D). The probabilities of death among children who live in the rural area (Fig. 1B) and delivered at home (Fig. 1F) were higher as compared to children living in urban and delivered at health institutions respectively.

### Model comparison

The study tried to fit different baseline hazard parametric distributional assumptions. Based on the information criteria Gompertz baseline distribution was the best fit model with gamma frailty distribution. The Log-logistic and Lognormal baseline hazard distribution model and the inverse Gaussian frailty distribution models did not converge. Therefore, Gompertz’s baseline distribution with gamma frailty distribution was the best fit model since it has the lowest AIC value (Table 5).

**Table 5:** Model comparison with different distributional assumptions

Model	Baseline hazard distribution	Frailty distribution	Frailty variance (θ, ***p***-value)	AIC	BIC	LLR
**Cox-model**	NA	Gamma				
**Shard frailty**	Exponential	Gamma	0.07, < 0.001	99,082.74	99,404.08	−49,510.37
**Shard frailty**	Weibull	Gamma	0.07, < 0.001	98,084.63	98,416.34	−49,010.32
**Shard frailty**	Gompertz	Gamma	0.07, < 0.001	96,693.04	97,024.74	−48,314.52
**Shard frailty**	Log-logistic	Gamma	The model didn’t converge			
**Shard frailty**	Lognormal	Gamma	The model didn’t converge			

Model with inverse Gaussian frailty distribution did not converge

*AIC* Akakian Information Criteria, *BIC* Bayesian Information Criteria, *LLR* Loglikelihood Ratio, *NA* Not Applicable

### Factors associated with under-five children mortality in Sub-Saharan Africa

To recognize the potential significant factors for under-five children mortality country-level parametric shared frailty survival model was fitted. The value of the shape parameter in the Gompertz baseline hazard distribution model was (ρ = −0.04, 95%CI: −0.043 – − 0.039). This negative value indicates that the hazard of death among under-five children had decreased exponentially as the age of under-five children increase. The dependency (heterogeneity) of under-five children in the same country estimated by the model was statistically significant with a value theta (θ = 0.07, 95% CI: 0.04-0.12), and the dependency within-country was τ = 3%.

After controlling country-level frailty, the results from Gompertz parametric baseline hazard distribution revealed the educational status of the husband/intimate partner, wealth status, residence, water source, sex of the child, preceding birth interval, birth order, birth size, and place of delivery were statistical predictors of under-five child survival.

The hazard of death among children who were born from rich and middle families was lower by 18% (AHR =0.82, 95% CI: 0.77-0.87) and 8% (AHR = 0.92, 95% CI: 0.87-0.97) respectively. The risks of death among under-five children were; residing in a rural area, used water from an unimproved source, and delivered at home. Those who reside in rural were 11% times more at risk of death when compared with urban dwellers (AHR = 1.06, 95% CI: 1.00-1.12). Those who had water from improved water sources had 19% times at risk of death when compared with those who had improved water sources (AHR = 1.11, 95%CI: 1.04-1.19). In the same way, those who were born at home had a 17% reduced risk of death when compared to those who are born in the health institutions (AHR = 1.17, 95% CI: 1.12-1.23).

The estimated hazard of death among female under-five children and not low birth weight were lowered by 7% as compared to male infants and low birth weight under-five children (AHR = 0.93, 95%CI: 0.90 – 0.97) and (AHR = 0.93 95%CI:0.89-0.97) respectively. The hazard of death among multiple-birth children was 2.1 times higher than singleton births (AHR = 2.11, 95% CI: 2.51 – 2.90). The estimated hazard among under-five children born less than 18 months and between 18 and 23 months preceding birth interval was a higher risk of death by 90 and 55% than children born greater than 24 months interval (AHR = 1.90, 95%CI: 1.78-2.04) and (AHR = 1.55, 95% CI:1.47-1.63) respectively. Children born from birth order of four and above was 3 times (AHR = 3.01, 95%CI: 2.85-3.19) higher risk of death as compared to the birth order of less than four (Table 6).

**Table 6:** Results of bivariate and multivariable parametric Gompertz distribution country-level shared frailty survival regression model among under-five children in Sub-Saharan Africa countries, 2021

Variable	Category	Children Status		CHR (95%CI)	AHR (95%CI)
		Alive	Dead		
**Age**	15-19	21,390	1720	1	1
	20-35	258,669	19,726	0.83 (0.77-0.88)*	0.97(0.78-1.200
	36-49	62,957	5775	0.89 (0.83-0.96)*	1.21(0.97-1.50
**Maternal educational status**	No education	142,583	13,911	1	1
	Primary	114,504	114,504	0.86 (0.83-0.90)*	0.97(0.92-1.03)
	Secondary and above	85,929	4925	0.53 (0.51-0.56)*	0.77(0.71-0.83)
**Husband educational status**	No education	111,662	11,196	1	1
	Primary	81,738	6078	0.85 (0.82-0.90)*	0.94 (0.89-1.01)
	Secondary and above	98,947	6563	0.63 (0.60-0.66*	0.87 (0.81-1.03)
**Wealth status**	Poor	162,455	14,339	1	1
	Middle	67,403	5452	0.82 (0.79-0.86)*	0.92 (0.87-0.97)*
	Rich	113,158	7430	0.60 (0.58-0.62*	0.82 (0.77-0.87)*
**Residence**	Rural	103,755	6904	1.44 (1.39-1.50)*	1.06 (1.00-1.12)*
	Urban	239,261	20,317	1	1
**Toilet facility**	No	116,714	10,480	1.18 (1.141.22)*	0.96 (0.91-1.01)
	Yes	226,198	16,723	1	1
**Water source**	Improved	78,491	4700	1	1
	Unimproved	264,471	22,514	1.40 (1.34-1.47)*	1.11 (1.04-1.19)*
**Sub-Saharan region**	East Africa	118,018	6890	0.88(0.55-1.42)	0.71(0.45-1.12)
	West Africa	72,755	5869	1.61(1.01-2.58)*	1.05 (0.68-1.63)
	Central Africa	141,121	13,776	1.44(0.86-2.41)	1.14(0.72-1.78)
	Southern Africa	11,122	686	1	1
**Sex**	Male	172,412	14,870	1	1
	Female	170,604	12,351	0.92 (0.89-0.95)*	0.93 (0.90-0.97)*
**Plurality**	Single	332,556	24,088	1	1
	Multiple	10,460	3133	2.54 (2.40-2.70)*	2.70 (2.51-2.90)*
**Preceding birth interval**	≥ 24 months	218,390	14,063	1	1
	18-23 months	33,235	3921	1.63 (1.60-1.71)*	1.55 (1.47-1.63)*
	< 18 months	16,956	3135	2.24 (2.13-2.36)*	1.90 (1.78-2.02)*
**Teenager pregnancy**	< 20 years	287,482	21,889	1.16 (1.12-1.21)*	0.95 (0.87-1.03
	≥20 years	55,534	5332	1	1
**Birth order**	≤three	193,511	14,081	1	1
	≥ four	149,505	13,140	1.19 (1.16-1.23)*	3.01 (2.85-3.19)*
**Birth size at birth**	Low birth weight	206,247	15,411	1	1
	Not low birth weight	120,712	7699	0.87 (0.84-0.90)*	0.93 (0.89-0.97)*
**Place of delivery**	Health facility	213,837	13,001	1	1
	Home	128,855	11,414	1.47 (1.41-1.52)*	1.17 (1.12-1.23)*
Gompertz distribution shape parameter gamma **(γ)**					**−0.04 (−0.043** – **−0.039)**
Frailty theta **(θ)**					**0.07(0.04-0.12)**
Frailty Kendall’s tau **(τ)**					**0.03**

*CHR* Crude Hazard Ratio, *AHR* Adjusted Hazard Ratio

*Significant at *P* < 0.05 levels

## Discussions

The estimation of child mortality is challenging for the great majority of developing countries without a well-functioning vital registration system due to issues with data quality (and for some countries data quantity) [16]. The Sub-Sahara region made significant development in increasing child survival for the past two decades. But the survival rates are still steady especially among newborns which indicates the region’s level of improvement in the quality of life [3, 9, 16]. And these rates are also important in identifying the directions for the public health program in the region.

In the current study, therefore, we tried to assess the survival status of under-five mortality and its determinants in Sub-Saharan Africa using the recent (2010-2018) Demographic and Health Survey data with country-based Shared frailty analysis. The result of this study indicated that out of 370,237 under-five children 27,221 (7.35%) were died before celebrating their fifth birth year. Children’s death depends on socio-demographic characteristics of the respondents, child characteristics, obstetric and environmental factors. Hence, this study revealed that the hazard of death among under-five children decreased exponentially as the age of under-five children increase. This means that the survival probability was very low among newborns. This finding is consistent with findings of other multi-country analyses [3, 7, 17]. Because newborns are exposed to prematurity, low birth weight, intrauterine growth retardation, and at an early age, since they are new for the external environments, newborns are susceptible to easily preventable child deaths such as birth trauma, asphyxia, hypothermia, sepsis, and lack of essential newborn care services. The higher number of births was associated with a greater risk of under-five mortality than their counterparts y. Studies have also shown a similarly elevated risk of under-five mortality among children born to mothers who gave more birth [3, 9, 18, 19]. This is because; higher parity is often associated with short-birth intervals, which influence under-five mortality risk through depletion of mother’s health and nutritional status, and premature birth [20]. Children from rich and middle economic status households experienced a lower risk of under-five mortality compared with households from poor economic status. In a similar vein, studies in 55 low resource countries using household assets index to measure wealth status found that under-five child mortality was significantly higher among households whose economic status is poor than households that had higher economic status [7, 21–23]. The effect of household wealth status on under-five children mortality may have operated through access to goods and services such as food, housing transportation, or financial access to care [24].

We also found evidence that the length of preceding birth interval was significantly associated with the risk of death before age of five. The hazard of death among under-five children born less than 18 months preceding birth interval was higher by 2 times than children born birth interval for more than 2 years. The risk of death is also higher by 1.5 times among children born from the birth interval between 18 and 23 months compared to a birth interval greater than 24 months. Thus, agreeing with the findings of some previous studies [3, 9, 16, 21, 22, 25]. A possible explanation is that a shorter length of the birth interval increases the risk of premature birth, low birth weight, maternal depletion syndrome, folate insufficiency, and newborn exposure to the susceptibility of infectious disease and in addition, it leads to competition in familial resource among children [26]. The risk of under-five mortality was slightly (by 7%) lower among female children as compared to male children. This has been explained by a sex difference in genetics and biological make-up that girls are advantage against many causes of death over boys [27, 28]. Excess male child mortality can be explained by biological factors (lower resistance to infection, higher risk of prematurity birth, difficult labor related to a larger average body size and head circumference), gender discrimination differential feeding and medical care practice, or response to HI related drugs [29, 30]. The size of children at birth was found to be significantly associated with higher under-five mortality.

Being low birth weight was a risk for child mortality. This evidence is also supported by other findings that small-sized children at birth have a higher risk of dying before they reach the fifth birthday [3, 21, 31]. This might be due to the immaturity of their organs which makes them find it more difficult to adapt to the external environment and independent life. Most premature babies are prone to have sepsis which is one of the major causes of under-five mortality [3, 32]. Furthermore, this study suggests that children from mothers who live in rural areas were significantly associated with high under-five mortality compared to those who live in the urban area. This concurs with previous studies in Cambodia and Nigeria [21, 25]. This is on the ground that those who live in the urban area have access to improved water supply, improved sanitation facility, unlimited health care access as well as other social and economic services [32, 33]. Findings have shown that children born other than health institutions have a 17% risk experience of under-five mortality compared to under-five children who were born in a health institution. Similar studies conducted in Pakistan [27], Indonesia [22], and Nigeria [21] showed that the place of delivery is significantly associated with childhood survival. Children born from mothers who have access to safe drinking water had a significant risk of under-five mortality.

This finding was supported by studies carried out in Cambodian [25], Nigeria [34], Egypt, and Eretria [35, 36]. The possible explanation for this finding is that children learn to crawl and walk and they experience exposure to pathogens that causes diarrhea from a variety of environmental sources, including contaminated water [37]. It is also households often use unimproved water to prepare weaning foods; thereby transmitting pathogens to children that causes diarrheal disease which has been resulted in high mortality [38, 39]. This study showed that the risk of death was 2.7 times higher among multiple birth under-five children as compared to singleton births. This evidence was similar to previous studies carried out in Unit states of America, Guinea Bissau, and Ethiopia [40–42].

The likely enlightenment for this evidence might be multi-fetal pregnancy and births lead to adverse pregnancy outcomes and child health, Another explanation might be parents with multiple births experience more anxiety, stress, and depression in the first year of life after birth than parents who had singletons birth. The probability of under-five mortality increases with total children ever born and this finding was consistent with preceding studies [32, 43, 44]. Also, an increase in the total number of children ever born could result in lack of care, low birth weight, premature births and heavy drain on the limited household resources as children have to compete for the little resources available for their survival. The likelihood of child survival decreased among children who are products of multiple births compared to their counterparts who were products of singletons. Low birth weight or competition for nutritional intake which occurs more among children who are products of multiple births could be a plausible explanation [44–46].

## Limitations

Data were collected cross-sectional at a different point in time by self-reported interview which might be prone to recall and social desirability bias. The drawback of the secondary nature of data was expected.

## Conclusions

The hazard of death among under-five children was higher at an early age than decrease as age advanced which means that the survival probability was very low among newborns. Wealth status, residence, water source, place of delivery, sex of the child, plurality, birth size, preceding birth interval, and birth order were the most predictor variables. In light of the above, we suggest that health care programs should be designed specifically to encourage a healthy family structure. Government and non-government organizations must work together to access safe water in the region and inspire maternal and child health services. The health care providers should intervene in the community to increase the health of the child and the health of the family as a whole.

## Data Availability

All the data sets are available on the hand of the corresponding author.

## Abbreviations

AHR: Adjusted Hazard Ratio
ANC: Antenatal care
DHS: Demographic and health survey
SSA: Sub Saharan Africa
GDB: Global Disease Burdon
SDGs: Sustainable Development Goals
WHO: World Health Organization
